# Distributed Event Triggering Algorithm for Multi-Agent System over a Packet Dropping Network

**DOI:** 10.3390/s21144835

**Published:** 2021-07-15

**Authors:** Ali Bemani, Niclas Björsell

**Affiliations:** Department of Electrical Engineering, Mathematics and Science, University of Gävle, 801 76 Gävle, Sweden; Ali.Bemani@hig.se

**Keywords:** wireless networked control system, distributed event-based state estimation, event-based triggering, packet drop

## Abstract

The availability of wireless networked control systems (WNCSs) has increased the interest in controlling multi-agent systems. Multiple feedback loops are closed over a shared communication network in such systems. An event triggering algorithm can significantly reduce network usage compared to the time triggering algorithm in WNCSs, however, the control performance is insecure in an industrial environment with a high probability of the packet dropping. This paper presents the design of a distributed event triggering algorithm in the state feedback controller for multi-agent systems, whose dynamics are subjected to the external interaction of other agents and under a random single packet drop scenario. Distributed event-based state estimation methods were applied in this work for designing a new event triggering algorithm for multi-agent systems while retaining satisfactory control performance, even in a high probability of packet drop condition. Simulation results for a multi-agent application show the main benefits and suitability of the proposed event triggering algorithm for multi-agent feedback control in WNCSs with packet drop imperfection.

## 1. Introduction

Recent modern communication, as well as its close combination with control, has direct consequences for the development of advanced wireless networked control systems (WNCSs) in industry [1]. Consensus problems for controlling multi-agent systems in WNCSs have been focused on by many researchers in recent years. This interest is due to the appearance of a diverse group of systems in engineering and science such as drone swarm, autonomous vehicles and hierarchical production in the industry like steel production or building automation, and many others [2,3,4]. Communication networks in these systems can be used as an essential tool for coordinating the agents together in interconnected systems to achieve a common goal of the whole system or improve the system’s overall performance.

Another active research area in WNCSs is the use of event-triggered control (ETC) instead of time-triggered control (TTC). In the TTC, data are periodically collected from the sensors and simultaneously sent to the controller. In contrast, in the ETC approach, data are not periodically collected. Instead, these are determined by an event-triggered system, in which the feedback loop is closed only when the states or control parameters satisfy a certain event condition [5].

Such event-triggered control strategies have recently been applied to multi-agent systems [2,3,4,5,6]. An important aspect of using ETC control strategies on multi-agent systems is that the subsystems should be coordinated without any extra components such as a coordinator. Furthermore, each agent has only limited access to the sensor information of other agents due to the limitations of communication capabilities. Based on these circumstances, a multi-agent system that is controlled through the wireless network requires a distributed control law and distributed control methods for designing the individual controllers for each agent [6].

**Figure 1:**
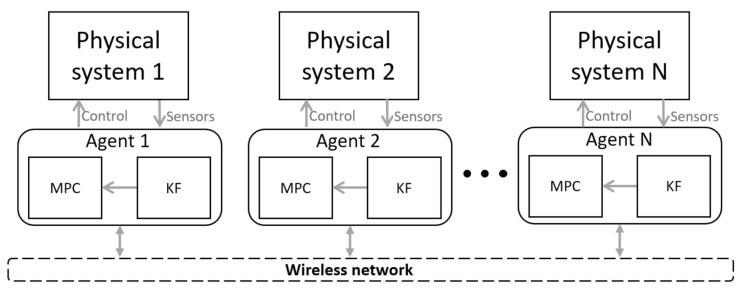
Cyber representation of a multi-agent system introduced in [7].

Figure 1 shows a cyber representation of a multi-agent system which was introduced in [7]. Agents are connected through a wireless network to implement a cooperative control with the multiple agents. The decentralized control method used to converge the consensus problem on each agent is the model predictive controller (MPC) with a state observer based on a Kalman filter (KF). Due to the nature of WNCSs, which can be severely affected by the imperfections of wireless communications, a predictive controller is one alternative to overcome the shortcomings of such systems.

The main problem in multi-agent systems is structuring the communication between the agents to ensure that they eventually take on a common state, track a synchronous path or achieve a joint task. We mainly focus on a multi-agent system in which they have mutual dependencies on each other, and they need to update their states in each process. However, due to the wireless network communication and the fact that the nodes use the shared bandwidth, the communication resource should be efficiently used. Thus, each agent should only use the communication resource when necessary. On the other hand, due to wireless communication, data transmission between the agents is often affected by environmental parameters such as packet drops, communication delay, and packet disorder, which should be considered in the controller’s design.

Developing such an event-based control system considering packet drop imperfection between the agents in a multi-agent system is the focus of this paper. The event-based method for dynamic state estimation has great importance in such systems. For example, Kalman filtering is one of the most widely used methods for state estimation in linear stochastic systems, so the Kalman filter (KF) implementation in the event-based approach with the presence of wireless communication imperfections like packet drops is of particular importance [8,9,10].

The main research question in this work is concerned with the design of the event triggering law using event-based state estimation in the presence of packet drop in WNCSs, which indicates when sending new data is necessary. This work uses distributed event-based state estimation (DEBSE) to estimate and predict the agents’ states in a multi-agent system. Similar approaches were developed for DEBSE without regarding the shortcomings of a wireless network in prior works [11,12,13,14,15].

The remainder of the paper is organized as follows. First, a brief review of the literature regarding this paper and related works are presented in Section 2. Then, the basic idea and related theory are discussed in Section 3. Section 4 is dedicated to defining the key variables and notation conventions that are used in this paper. Subsequently, Section 5 describes the system model of the multi-agent system in which the agents have interconnections in their process with each other and formulate the event triggering solution. Then, Section 6 is dedicated to illustrating the behavior of the proposed event triggering algorithm in one application and divided into two parts: modeling a vehicle platooning as an application and the simulation platform. Finally, Section 7 demonstrates the simulation results and the benefits of the proposed event triggering algorithm.

## 2. Related Work

The importance of a network control system and achieving high-performance control on a resource-limited system has been recently pointed out by many researchers. For example, this can be seen in [2,3,4,8] for the control of a multi-agent system via event-based communication, in [9,10,11,12,13] for Kalman filtering as an estimator to design an event-based state estimation system, and in [16,17,18,19,20] for optimal state estimation in the presence of packet drop in the networked control system.

State estimations and predictions are the main components of an event-based triggering system, which mostly compute the states in the sense of minimum mean square error [21,22]. Various studies have been proposed to develop a different type of Kalman filtering in the presence of wireless communication imperfection, for example, in the presence of data packet drops [16,23,24,25]. Due to the distributed systems in WNCSs, recent studies have been focused on the study of distributed Kalman filtering, where each agent in a WNCS can compute local states’ estimation and prediction via Kalman filtering based on its own sensor measurements and the information received from other agents [26,27,28,29,30,31]. Several algorithms based on the consensus problem were proposed in these research papers. Most of the algorithms for the Gaussian systems are based on different types of KF, which are used to design new methods for the event-based triggering algorithm in WNCS.

In the ETC approach, continuous sampling from the agents has always been required to determine whether the ET condition has been reached. In order to eliminate such limitations, the concept of self-triggered control has been proposed [32,33]. With this approach, it is possible to predict the need for sampling in a future instant and determine the next triggering time at the previous trigger. However, in both approaches, the trigger interval must have a lower bound to exclude the Zeno behavior. It is a phenomenon in a hybrid system and happens when an infinite number of discrete transitions occur in a finite time interval. To overcome such problems, periodic event-triggered control is proposed for the synchronization for a discrete-time linear stochastic dynamic system. Various research studies on periodic event triggering for consensus problems in multi-agent systems were discussed in [34,35].

Various control design methodologies have been proposed for the consensus problem of networked multi-agent systems. For example, this can be seen in [36,37] for the state feedback control of a multi-agent system in the event of data packet drops, and in [38,39] for a distributed model predictive control algorithm for heterogeneous multi-agent systems with directional and unidirectional topology. Wang, in [40], presented an event-triggered consensus strategy with state feedback for a linear multi-agent system, where random packet losses were taken into account.

To the best of our knowledge, few researchers have proposed DEBSE with a state feedback control of multi-agent systems and none of the mentioned references considered the new approach which is the subject of this article, where the event triggering algorithm is formulated as two parallel decision problems with the use of state estimation and prediction. The concept of triggering decisions in the presence of data packet drops in networked multi-agent systems is novel.

## 3. Basic Idea and Related Theory

**Figure 2:**
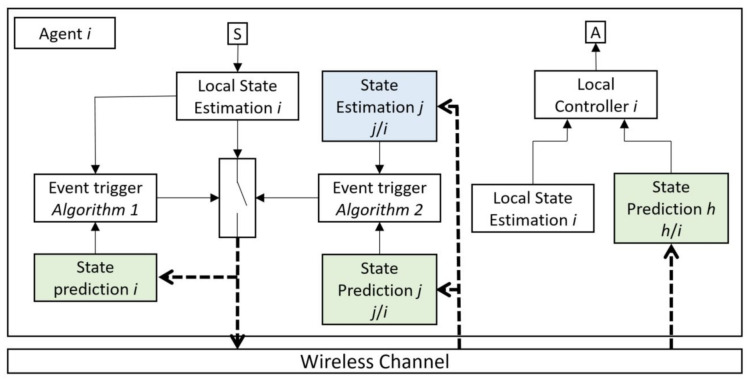
Event triggering algorithm is based on DEBSE for each agent i=1,…,N in a multi-agent system. The dynamics of each physical plant are connected to its agent via sensors (S) and actuators (A).

The main idea of DEBSE in this work is to use the model-based estimation and prediction of other agents, which can be used in the triggering algorithm to broadcast new information to the other agents and prevent continuous data broadcasting. Figure 2 shows one of the agents in the multi-agent system.

The agent *i*’s dynamic will be coupled with all or a subset of other agents as follows:

(1) $$x_{k}^{i}=A_{i}x_{k-1}^{i}+B_{i}u_{k-1}^{i}+w_{k}^{i}+\sum_{h\in N_{N},h\neq \left[i\right]}N_{h}{\overset{ˇ}{x}}_{k-1}^{h}\;,$$

where $N_{h}$ is the interaction matrix between the agents in the multi-agent system, $x_{k}^{i}\in R^{n_{x}}$ denotes the state, ${\overset{ˇ}{x}}_{k-1}^{h}\in R^{n_{x}}$ denotes the remote state prediction of other agents in agent *i*, $u_{k}^{i}\in R^{n_{u}}$ denotes the input, $w_{k}^{i}\in R^{n_{x}}$ denotes process noise, and $A_{i},B_{i}$ denote the dynamic system parameters for agent *i*. A discrete-time linear process with Gaussian noise is considered for each agent and the interaction between them.

An event triggering decision is based on two parallel algorithms, the first one based on comparing the state prediction of agent *i* with the state estimation of this agent, and the second one is based on comparing the state prediction with the state estimation of other agents which have communication with agent *i* (state prediction *j* and state estimation *j*).

The first event triggering algorithm is designed to check the accuracy of prediction without knowing whether the packet drop has happened. Furthermore, the second event triggering algorithm examines the difference between the estimation and prediction of other agents in agent *i* which interact with agent *i* in their process. In this way, it could be checked whether the discrepancy between the estimation and prediction increases, which indicates that in the previous broadcasting of agent *i*, a packet drop occurred, and now an event trigger is needed to improve the prediction of agent *i* in other agents. In this proposed event triggering algorithm, a few extra instances of communication between the agents is required, but it is assumed that when an event is triggered in one agent, its states are sent not only to the agents that interact with them, but also to the agents that receive interaction from them. In this way, additional communications will be limited, and sending states work like group broadcasting. This extra communication load could be estimated based on the multi-agent system’s topology and the interaction between the agents.

The main contribution of this study is the proposal of a new event triggering mechanism for a distributed multi-agent system in the presence of packet drop imperfection to preserve the system performance under such conditions. The event triggering mechanism is derived as parallel event triggering compared with the general event triggering for the distributed system under conditions with a high probability of packet drop.

### DEBSE Architecture

The main components of the DEBSE architecture in this configuration are organized as follows:

1. Local State Estimation agent *i*: A KF state estimator is used to estimate all states of the agent *i* based on the dynamics model of this agent, the measured values from its sensors, and the other agents’ state prediction that interacts with this agent.
2. Local State Prediction agent *i*: A KF state predictor considering that the packet drop is used to predict all states of agent *i* based on the dynamics model of this agent, the last state values that buffered in the previous ET mechanism, and the other agents’ state prediction that interacts with this agent.
3. State Estimation agent *j/i*: A KF state estimator considering that the packet drop is used to estimate all states of the agent *j* based on the dynamics model of this agent and its state values, which is received by agent *i* in the previous ET broadcasting of agent *j*. Based on this scenario, in a distributed multi-agent system, the dynamic model of agent *j* is affected by the state values of agent *i*, which is attainable in the estimation of agent *j*. The agent *j* could be all or a subset of agents based on the agents’ dynamics models.
4. State Prediction agent *j/i*: This state predictor is the same as the state estimator of agent *j*, except that the state values of agent *i* are not attainable in this prediction. Due to the possibility of packet loss, access or lack of access to the information of the state values of agent *i* is not clear for agent *j* and therefore is not included in the predictor.
5. Event Trigger Algorithm 1: The first decision algorithm decides when the ET needs to be activated, and a new update is sent to all agents. This is the first part of a parallel event triggering (PET) algorithm. In this ET, the local state estimation is compared with the local state prediction, which is the predictor of its own behavior in other agents.
6. Event Trigger Algorithm 2: The second decision algorithm, similar to the first one, decides when the ET needs to be activated, but this ET is not based on the local estimation and prediction. This is the second part of the PET algorithm. In this ET, the state estimation of agent j is compared with the state prediction of its agent. The number of ET in this scenario could be a part of the whole number of agents that their dynamic model is affected by the state values of the agent *i*.
7. State Prediction agent *h*: A KF state predictor considering that the packet drop is used to predict all states of agent *h*. The agent *h* could be all or a subset of agents that have an interconnection with the dynamic model of the agent *i*.
8. Local Control agent *i*: The local controller can be designed for each agent independently, and it decides for its actuator. However, for the coordination problem, it needs the information from its state estimation and all other agents’ prediction (agent *h*) that have an interconnection with the agent *i*. The optimal controller for each agent is given as the solution for coordination or consensus problems by minimizing a quadratic cost function as an optimal linear quadratic regulator (LQR). The control decision in this agent is based on its state estimation and prediction of all other agents influencing this agent (state prediction *h*).

This structure presents the design of a robust distributed state feedback controller for a multi-agent system in which each agent has all the necessary information to take the control decision. The dynamics of each agent are subjected to external disturbances and under random packet drop in network communication between the agents. The key benefit of this structure is the improvement of the system performance in the presence of packet drop imperfection in wireless communication, in such a multi-agent system. Furthermore, improving the use of communication resources is another benefit of this structure. ET has been developed in DEBSE to reduce the need for feedback and preserve a certain level of performance in such a system.

## 4. Definitions and Preliminaries

The following notation conventions and key variables will be used in this paper. $A_{i},B_{i},C_{i},Q_{i},R_{i}$ denote the dynamic system parameters for each agent. N denotes the set of all non-negative integers, $N_{>0}$ denotes the set of all positive integers, R denotes the field of all real numbers, and for N∈N, we write the set {1,2,…,N} as $N_{N}$. By ∥·∥, we donate the Euclidean norm. $F_{i}$ is the optimal feedback gain corresponding to agent *i* and $u_{k}^{i}$ is the control input for each agent. $x_{k}^{i}$ is the state of agent *i*, ${\hat{x}}_{k}^{i}$ is the KF state estimate of agent *i*, ${\overset{ˇ}{x}}_{k}^{i}$ is the remote state estimate of agent *i* with the use of the KF predictor, ${\hat{x}}_{k}^{j/i}$ is the remote state estimation of agent *j* through agent *i*’s process, and ${\overset{ˇ}{x}}_{k}^{j/i}$ is the remote state prediction of agent *j* through agent *i*. $\Xi_{k}^{i}$ denotes all information gathered from sensors on agent *i*:

(2) $$\Xi_{k}^{i}=\left\{y_{1}^{i},\ldots ,y_{k}^{i},u_{1}^{i},\ldots ,u_{k-1}^{i}\right\}.$$

Let $\gamma_{k}^{i}\in \left\{0,1\right\}$ be a communication decision variable for agent *i* such that $\gamma_{k}^{i}=1$ if and only if ${\hat{x}}_{k}^{i}$ is to be transmitted to all other agents at time *k*, $\Gamma_{k}^{i}=\left\{\gamma_{1}^{i},\ldots ,\gamma_{k}^{i}\right\}$ is the set of communication decision. Therefore, $I_{k}^{i}$ denotes the information set available to the agent *i* at time *k*:

(3) $$I_{k}^{i}=\left\{y_{1}^{i},\ldots ,y_{k}^{i},u_{1}^{i},\ldots ,u_{k-1}^{i},\gamma_{1}^{i},\ldots ,\gamma_{k}^{i}\right\}.$$

For ease of notation, key variables from this and the later sections are summarized in Table 1.

Based on $\Xi_{k}^{i}$, the local state estimates and error covariance are defined by

(4) $$\begin{array}{cc} {\hat{x}}_{k|k-1}^{i} & ≜E\left[x_{k}|\Xi_{k-1}^{i}\right], \end{array}$$

(5) $$\begin{array}{cc} P_{k|k-1}^{i(E)} & ≜E\left[\left(x_{k}-{\hat{x}}_{k|k-1}^{i}\right){\left(x_{k}-{\hat{x}}_{k|k-1}^{i}\right)}^{T}|\Xi_{k-1}^{i}\right], \end{array}$$

(6) $$\begin{array}{cc} {\hat{x}}_{k|k}^{i} & ≜E\left[x_{k}|\Xi_{k}^{i}\right], \end{array}$$

(7) $$\begin{array}{cc} P_{k|k}^{i(E)} & ≜E\left[\left(x_{k}-{\hat{x}}_{k|k}^{i}\right){\left(x_{k}-{\hat{x}}_{k|k}^{i}\right)}^{T}|\Xi_{k}^{i}\right], \end{array}$$

and based on $I_{k}^{i}$, the state prediction and error covariance are defined by

(8) $$\begin{array}{cc} {\overset{ˇ}{x}}_{k|k-1}^{i} & ≜E\left[x_{k}|I_{k-1}^{i}\right], \end{array}$$

(9) $$\begin{array}{cc} P_{k|k-1}^{i(P)} & ≜E\left[\left(x_{k}-{\overset{ˇ}{x}}_{k|k-1}^{i}\right){\left(x_{k}-{\overset{ˇ}{x}}_{k|k-1}^{i}\right)}^{T}|I_{k-1}^{i}\right], \end{array}$$

(10) $$\begin{array}{cc} {\overset{ˇ}{x}}_{k|k}^{i} & ≜E\left[x_{k}|I_{k}^{i}\right], \end{array}$$

(11) $$\begin{array}{cc} P_{k|k}^{i(P)} & ≜E\left[\left(x_{k}-{\overset{ˇ}{x}}_{k|k}^{i}\right){\left(x_{k}-{\overset{ˇ}{x}}_{k|k}^{i}\right)}^{T}|I_{k}^{i}\right]. \end{array}$$

In the instance when $\gamma_{k}^{i}=1$, agent *i* broadcasts its local state estimate ${\hat{x}}_{k}^{i}$ over a packet dropping channel to all other agents. Let $\nu_{k}^{j/i}$ be random variables such that $\nu_{k}^{j/i}=1$ if the agent *j*’s states broadcasting at time *k* is successfully received by agent *i*, and $\nu_{k}^{j/i}=0$ otherwise. We will assume that {ν} is i.i.d. Bernoulli with:

(12) $$P\left(\nu_{k}^{j/i}=1\right)=\lambda_{j/i}\in \left(0,1\right)\;\forall k\geq 0,$$

we assume that $\nu_{k}^{j/i}$ and $\nu_{k}^{i/j}$ are independent of each other ∀i≠j, however, $\lambda_{j/i}=\lambda_{i/j}$ is allowed.

Based on packet drop information, we will define the information set available to the agent *i* which comes from the agent *j* at time *k* as

(13) $$I_{k}^{j/i}=\left\{\gamma_{1}^{j}\nu_{1}^{j/i}{\hat{x}}_{1|1}^{j},\ldots ,\gamma_{k}^{j}\nu_{k}^{j/i}{\hat{x}}_{k|k}^{j},u_{1}^{i},\ldots ,u_{k-1}^{i}\right\},$$

and the state estimation of agent *j* in agent *i* and its error covariance are defined as

(14) $$\begin{array}{cc} {\hat{x}}_{k|k-1}^{j/i} & ≜E\left[x_{k}^{j}|I_{k-1}^{i},I_{k-1}^{j/i}\right], \end{array}$$

(15) $$\begin{array}{cc} P_{k|k-1}^{j/i(E)} & ≜E\left[\left(x_{k}^{j}-{\hat{x}}_{k|k-1}^{j/i}\right){\left(x_{k}^{j}-{\hat{x}}_{k|k-1}^{j/i}\right)}^{T}|I_{k-1}^{i},I_{k-1}^{j/i}\right], \end{array}$$

(16) $$\begin{array}{cc} {\hat{x}}_{k|k}^{j/i} & ≜E\left[x_{k}^{j}|I_{k}^{i},I_{k}^{j/i}\right], \end{array}$$

(17) $$\begin{array}{cc} P_{k|k}^{j/i(E)} & ≜E\left[\left(x_{k}^{j}-{\hat{x}}_{k|k}^{j/i}\right){\left(x_{k}^{j}-{\hat{x}}_{k|k}^{j/i}\right)}^{T}|I_{k}^{i},I_{k}^{j/i}\right], \end{array}$$

and the state prediction of agent *j* in agent *i* and its error covariance are defined as follows:

(18) $$\begin{array}{cc} {\overset{ˇ}{x}}_{k|k-1}^{j/i} & ≜E\left[x_{k}^{j}|I_{k-1}^{j/i}\right], \end{array}$$

(19) $$\begin{array}{cc} P_{k|k-1}^{j/i(P)} & ≜E\left[\left(x_{k}^{j}-{\overset{ˇ}{x}}_{k|k-1}^{j/i}\right){\left(x_{k}^{j}-{\overset{ˇ}{x}}_{k|k-1}^{j/i}\right)}^{T}|I_{k-1}^{j/i}\right], \end{array}$$

(20) $$\begin{array}{cc} {\overset{ˇ}{x}}_{k|k}^{j} & ≜E\left[x_{k}^{j}|I_{k}^{j/i}\right], \end{array}$$

(21) $$\begin{array}{cc} P_{k|k}^{j/i(P)} & ≜E\left[\left(x_{k}^{j}-{\overset{ˇ}{x}}_{k|k}^{j/i}\right){\left(x_{k}^{j}-{\overset{ˇ}{x}}_{k|k}^{j/i}\right)}^{T}|I_{k}^{j/i}\right]. \end{array}$$

The decision variables $\gamma_{k}^{i}$ are computed for agent *i* based on the self information set, available to agent *i*$\left(I_{k}^{i}\right)$ and the information set received from other agents (based on information available at time *k*− 1) over a packet dropping channel between agents $\left(I_{k}^{j/i}\right)$. Furthermore, the local controller agent *i* computes the optimal feedback gain based on these data $\left(I_{k}^{i}\right),\left(I_{k}^{h/i}\right)$, which shows that there is an interaction between agents *i* and *h*.

## 5. System Model and Fundamental Triggering

This section will formulate the event triggering solution as two threshold algorithms for each agent in multi-agent systems. When the event is triggered, the local state estimation shall be transmitted to other agents. We wish to jointly design the transmission decisions and control signal for each agent to solve an optimal feedback problem.

**Figure 3:**
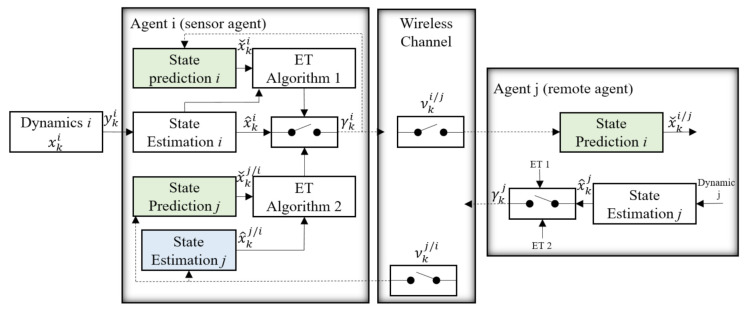
Event triggering problem. The sensor agent *i* broadcasts its local state estimate ${\hat{x}}_{k}^{i}$ in case of a positive triggering decision ($\lambda_{k}^{i}=1$) and is received by agent *j* with the probability of $\nu_{i/j}$.

We considered the configuration of a multi-agent system in Figure 3, which was reduced to the main components for a precise analysis. Agent *i*, which is called a sensor agent in this step, broadcasts its local state estimation over the wireless network with a probability of packet drop in the case of a positive triggering decision ($\lambda_{k}^{i}=1$). These data are received by agent *j* with the probability of $\lambda_{i/j}$. Agent *j*, which is called the remote agent, stands representative for any of the agents in a multi-agent system in this configuration and requires the information from agent *i* to solve the local control problem. Furthermore, agent *j* broadcasts its local state estimates when the event is triggered, and the data are received by agent *i* with the probability of $\lambda_{j/i}$. These data are used to check the accuracy of state estimation of agent *j* through agent *i* in the second event triggering algorithm. To the best of the authors’ knowledge, event triggering with this parallel mechanism is a new concept in both estimation and control in an industrial environment with a high probability of packet drops. Previous works focused on the estimation and prediction of agent *i* without considering whether a packet drop has happened.

In the following, we introduced the main components of Figure 3 and precisely formulated the event triggering problem.

### 5.1. Process Dynamics

We considered a discrete-time linear process with Gaussian noise for each agent *i* with its interactions with other agents:

(22) $$\begin{array}{cc} x_{k}^{i} & =A_{i}x_{k-1}^{i}+B_{i}u_{k-1}^{i}+w_{k}^{i}+\sum_{h\in N_{N},h\neq \left[i\right]}N_{h}{\overset{ˇ}{x}}_{k-1}^{h}\;, \end{array}$$

(23) $$\begin{array}{cc} y_{k}^{i} & =C_{i}x_{k}^{i}+v_{k}^{i}\;, \end{array}$$

where $x_{k}^{i}\in R^{n_{x}}$ denotes the state, ${\overset{ˇ}{x}}_{k-1}^{h}\in R^{n_{x}}$ denotes the remote state prediction of other agents in agent *i*, $u_{k}^{i}\in R^{n_{u}}$ denotes the input, $w_{k}^{i}\in R^{n_{x}}$ denotes process noise which is i.i.d. Gaussian with zero mean and covariance $Q_{i}$, $y_{k}^{i}\in R^{n_{y}}$ denotes the sensor measurements, and $v_{k}^{i}\in R^{n_{y}}$ denotes the measurement noise which is Gaussian with zero mean and covariance $R_{i}$. The random variables $x_{k}^{i}$, $w_{k}^{i}$, and $v_{k}^{i}$ are assumed to be mutually independent. For the local estimation, $R_{i}$ can be found by removing the mean value from the measurements. The calculation for $Q_{i}$ is not straightforward. Parameter uncertainties can be modeled as the process noise. For the remote estimation, the calculation of $R_{i}$ and $Q_{i}$ is the same as the previous method, considering that the measurement data can be lost due to the message drop via wireless communication. Here, it will be appropriate to define the auxiliary variable $\psi_{k}^{i}=\sum_{h\in N_{N},h\neq \left[i\right]}N_{h}{\overset{ˇ}{x}}_{k}^{h}$ for the different equations.

### 5.2. Local State Estimation Agent *i*

Local state estimator on agent *i* has access to all inputs and information gathered from sensors $\Xi_{k}^{i}$. The local state estimates and error covariance for this agent can be computed using the standard Kalman filtering equation:

(24) $$\begin{array}{cc} {\hat{x}}_{k|k-1}^{i} & =A_{i}{\hat{x}}_{k-1|k-1}^{i}+B_{i}u_{k-1}^{i}+\psi_{k-1}^{i}\;, \end{array}$$

(25) $$\begin{array}{cc} P_{k|k-1}^{i} & =A_{i}P_{k-1|k-1}^{i}A_{i}^{T}+Q_{i}\;, \end{array}$$

(26) $$\begin{array}{cc} {\hat{x}}_{k|k}^{i} & ={\hat{x}}_{k|k-1}^{i}+K_{k}^{i}\left(y_{k}^{i}-C_{i}{\hat{x}}_{k|k-1}^{i}\right)\;, \end{array}$$

(27) $$\begin{array}{cc} P_{k|k}^{i} & =\left(I-K_{k}^{i}C_{i}\right)P_{k|k-1}^{i}\;, \end{array}$$

where $K_{k}^{i}=P_{k-1|k-1}^{i}C_{i}^{T}{\left(C_{i}P_{k-1|k-1}^{i}C_{i}^{T}+R_{i}\right)}^{-1}$.

### 5.3. Local Control Agent *i*

We want to optimize the following cost function for the control and communication algorithm for each agent where the optimization algorithm calculates the communication decision $\left\{\gamma_{k}^{i}\right\}$ and control signals $\left\{u_{k}^{i}\right\}$ as follows:

(28) $$\min_{\left\{\gamma_{k}^{i},u_{k}^{i}\right\}}E\left[\sum_{k=0}^{M-1}x_{k}^{i}{}^{T}W_{i}x_{k}^{i}+u_{k}^{i}{}^{T}U_{i}u_{k}^{i}+\gamma_{k}^{i}\delta_{k}^{i}+\left(1-\gamma_{k}^{i}\right)\left(E_{1|k}^{i}+E_{2|k}^{i}\right)\right].$$

The estimation cost $E_{1}^{i}$ is used to measure the discrepancy between the estimation and prediction of agent *i* which we write as the quadratic norm:

(29) $$E_{1|k}^{i}≜{∥{\hat{x}}_{k}^{i}-{\overset{ˇ}{x}}_{k}^{i}∥}^{2},$$

and the estimation cost $E_{2}^{j}$ is used to measure the discrepancy between the estimation and prediction of agent *j* in *i*, which we write as follows:

(30) $$E_{2|k}^{j}≜{∥{\hat{x}}_{k}^{j/i}-{\overset{ˇ}{x}}_{k}^{j/i}∥}^{2}.$$

The estimation cost *E*_1_ is related to the first event triggering algorithm, and *E*_2_ is associated with the second one.

The matrices $W_{i}\geq 0$, $U_{i}\geq 0$ are the parameters weighting for the LQR control, scalar $\delta_{k}^{i}\geq 0$ is a design parameter to evaluate the accuracy of estimation and prediction for the event-triggered condition for agent *i*, $E_{1|k}^{i}$ is the estimation cost for agent *i*, and $E_{2|k}^{i}≜\sum_{j\in N_{N}\backslash \left[i\right]}E_{2|k}^{j}$ is the total remote estimation cost of other agents through agent *i*. The fact that this optimization problem is mentioned in [8] and used for one process proves that the design of $\left\{\gamma_{k}^{i}\right\}$ and $\left\{u_{k}^{i}\right\}$ can be separated from each other. Furthermore, we can solve the LQR problem for the first part to calculate the optimal feedback gain for each agent:

(31) $$\min_{\left\{u_{k}^{i}\right\}}E\left[\sum_{k=0}^{M-1}\left(x_{k}^{i}{}^{T}W_{i}x_{k}^{i}+u_{k}^{i}{}^{T}U_{i}u_{k}^{i}\right)+x_{N}^{i}{}^{T}W_{i}x_{N}^{i}\right],$$

thus, the optimal solution to this problem is of the form:

(32) $$u_{k-1}^{i}=-{\left(B_{i}^{T}P_{k-1}^{i}+B_{i}\right)}^{-1}B_{i}^{T}P_{k-1}^{i}A_{i}≜-F_{i}{\hat{x}}_{k-1}^{i},$$

where, as mentioned before, in (24), ${\hat{x}}_{k-1}^{i}$ is the state estimation of the agent *i* with its interaction with other agents in its dynamic. Furthermore, the minimization problem in (31) could be reconfigured as a tracking problem to find control law $u_{k}^{i}$ in such that:

(33) $$\begin{array}{c} \lim_{k\to \inf}∥x_{k}^{i}-x_{ref,k}^{i}∥=0, \end{array}$$

(34) $$\begin{array}{c} u_{k}^{i}=F_{i}\left(x_{k}^{i}-x_{ref,k}^{i}\right), \end{array}$$

where $x_{ref}^{i}$ is the desired states for agent *i*. The second part of the optimization problem for event triggering will be subsequently introduced.

### 5.4. Remote State Estimation Agent *i* (State Prediction Agent *i*)

When an event is triggered according to both event triggering algorithms in Figure 3, the sensor agent communicates its local estimate ${\hat{x}}_{k}^{i}$ to all the remote agents, but due to the possibility of packet loss, agent *i* has no information with regard to receiving its estimate, for example, by agent *j*. Therefore, it can be easily shown that the state prediction process of agent *i* in itself can be computed by

(35) $$\begin{array}{c} \begin{array}{c} {\overset{ˇ}{x}}_{k|k}^{i}=\left\{\begin{array}{cc} A_{i}{\overset{ˇ}{x}}_{k-1|k-1}^{i}+B_{i}u_{k-1}^{i}+\psi_{k-1}^{i} & ,\gamma_{k}^{i}=0\\ {\hat{x}}_{k|k}^{i} & ,\gamma_{k}^{i}=1 \end{array}\right. \end{array}, \end{array}$$

that is, when the event is not triggered, the predictor in agent *i* predicts its states according to the process model and the control input, which are calculated in (32). Furthermore, the state prediction of the agent *i* in *j* can be computed by

(36) $$\begin{array}{c} \begin{array}{c} {\overset{ˇ}{x}}_{k|k}^{i/j}=\left\{\begin{array}{cc} A_{i}{\overset{ˇ}{x}}_{k-1|k-1}^{i/j}+B_{i}{\overset{ˇ}{u}}_{k-1}^{i}+\psi_{k-1}^{i} & ,\gamma_{k}^{i}\nu_{k}^{i/j}=0\\ {\hat{x}}_{k|k}^{i} & ,\gamma_{k}^{i}\nu_{k}^{i/j}=1 \end{array}\right. \end{array}, \end{array}$$

and that is when the packet is dropped, or the event is not triggered, the predictor in agent *j* predicts the states of agent *i* according to that process model, and the control input is also predicted with the use of state prediction as follows:

(37) $$\begin{array}{c} {\overset{ˇ}{u}}_{k-1}^{i}≜-F_{i}{\overset{ˇ}{x}}_{k-1}^{i/j}. \end{array}$$

In this scenario, when a new agent is added to the network, it should broadcast all its model parameters to other agents, then each agent can predict the optimal feedback of other agents and use in (37). The state prediction of agent *i* in the sensor side (32) is used in the first event triggering algorithm, as shown in Figure 3.

### 5.5. State Estimation and Prediction of Agent *j* in *i*

To check the accuracy of the agent *i*’s state prediction in remote agents, we need to have another estimation and prediction of agent *j*’s state on the sensor side. This prediction and estimation are used for the second event triggering algorithm. This event triggering algorithm shows that the prediction of agent *i* in *j* is not sufficiently accurate due to the possibility of packet loss, and a new event needs to be triggered in agent *i*. For this purpose, the previous scenario for the remote state estimation agent *i* is repeated here. Furthermore, the estimator knows that agent *j* has some interaction within the process model with agent *i*. Hence, the estimator on the sensor side has access to this information and uses them in its estimation model. The state estimation of agent *j* in *i* can be computed as follows.

1. Time update:
  (38) $$\begin{array}{cc} {\hat{x}}_{k|k-1}^{j/i} & =A_{j}{\hat{x}}_{k-1|k-1}^{j/i}+B_{j}{\overset{ˇ}{u}}_{k-1}^{j/i}+N_{i}{\hat{x}}_{k-1|k-1}^{i}+\sum_{h\in N_{N},h\neq \left[j\right],\left[i\right]}\gamma_{k}^{h}\nu_{k}^{h/i}N_{h}{\overset{ˇ}{x}}_{k-1}^{h/i}, \end{array}$$
  (39) $$\begin{array}{cc} P_{k|k-1}^{j/i(E)} & =A_{j}P_{k-1|k-1}^{j/i(E)}A_{j}^{T}+N_{i}P_{k-1|k-1}^{i(E)}N_{i}^{T}+\sum_{h\in N_{N},h\neq \left[j\right],\left[i\right]}\gamma_{k}^{h}\nu_{k}^{h/i}N_{h}P_{k-1|k-1}^{h/i(E)}{N_{h}}^{T}+Q_{j}, \end{array}$$
2. Measurements update:
  (a) if $\gamma_{k}^{j}\nu_{k}^{j/i}=1$
    (40) $$\begin{array}{cc} K_{k}^{j/i} & =P_{k-1|k-1}^{j/i(E)}C_{j}^{T}{\left(C_{j}P_{k-1|k-1}^{j/i(E)}C_{j}^{T}+R_{j}\right)}^{-1}, \end{array}$$
    (41) $$\begin{array}{cc} {\hat{x}}_{k|k}^{j/i} & ={\hat{x}}_{k|k-1}^{j/i}+K_{k}^{j}\left({\tilde{x}}_{s|\gamma_{s}^{j}\nu_{s}^{j/i}=1}^{j/i}-C_{i}{\hat{x}}_{k|k-1}^{j/i}\right), \end{array}$$
    (42) $$\begin{array}{cc} P_{k|k}^{j/i(E)} & =\left(I-K_{k}^{j/i}C_{j}\right)P_{k|k-1}^{j/i(E)}, \end{array}$$
  (b) if $\gamma_{k}^{j}\nu_{k}^{j/i}=0$
    (43) $$\begin{array}{cc} {\hat{x}}_{k|k}^{j/i} & ={\hat{x}}_{k|k-1}^{j/i}, \end{array}$$
    (44) $$\begin{array}{cc} P_{k|k}^{j/i(E)} & =P_{k|k-1}^{j/i(E)}, \end{array}$$

where ${\tilde{x}}_{s|\gamma_{s}^{j}\nu_{s}^{j/i}=1}^{j/i}$ is the latest state of agent *j* which is correctly received by agent *i* from agent *j*’s broadcasting in time s≤k.

The predictor of agent *j* in *i* is designed in such a way that has no access to any information of interaction processes between other agents, especially agent *i*. Therefore, the interaction part is removed in its calculation. This means that if a packet drop has occurred in the previous broadcasting of agent *i*, then the prediction of agent *j* in *i* loses its accuracy in front of the estimation of agent *j* in *i* which has access to the information of agent *i*. This deviation will be used in the second event triggering algorithm. With this definition, the prediction of agent *j* in *i* has not any access to the information of agent *i* and becomes what is described as follows.

1. Time update:
  (45) $$\begin{array}{cc} {\overset{ˇ}{x}}_{k|k-1}^{j/i} & =A_{j}{\overset{ˇ}{x}}_{k-1|k-1}^{j/i}+B_{j/i}{\overset{ˇ}{u}}_{k-1}^{j/i}+\sum_{h\in N_{N},h\neq \left[j\right],\left[i\right]}\gamma_{k}^{h}\nu_{k}^{h/i}N_{h}{\overset{ˇ}{x}}_{k-1}^{h/i} \end{array}$$
  (46) $$\begin{array}{cc} P_{k|k-1}^{j/i(P)} & =A_{j}P_{k-1|k-1}^{j/i(P)}A_{j}^{T}+\sum_{h\in N_{N},h\neq \left[j\right],\left[i\right]}\gamma_{k}^{h}\nu_{k}^{h/i}N_{h}P_{k-1|k-1}^{h/i(P)}{N_{h}}^{T}+Q_{j} \end{array}$$
2. Measurements update:
  (a) if $\gamma_{k}^{j}\nu_{k}^{j/i}=1$
    (47) $$\begin{array}{cc} K_{k}^{j/i} & =P_{k-1|k-1}^{j/i(P)}C_{j}^{T}{\left(C_{j}P_{k-1|k-1}^{j/i(P)}C_{j}^{T}+R_{j}\right)}^{-1} \end{array}$$
    (48) $$\begin{array}{cc} {\overset{ˇ}{x}}_{k|k}^{j/i} & ={\overset{ˇ}{x}}_{k|k-1}^{j/i}+K_{k}^{j/i}\left({\tilde{x}}_{s|\gamma_{s}^{j}\nu_{s}^{j/i}=1}^{j/i}-C_{i}{\overset{ˇ}{x}}_{k|k-1}^{j/i}\right) \end{array}$$
    (49) $$\begin{array}{cc} P_{k|k}^{j/i(P)} & =\left(I-K_{k}^{j/i}C_{j}\right)P_{k|k-1}^{j/i(P)} \end{array}$$
  (b) if $\gamma_{k}^{j}\nu_{k}^{j/i}=0$
    (50) $$\begin{array}{cc} {\overset{ˇ}{x}}_{k|k}^{j/i} & ={\overset{ˇ}{x}}_{k|k-1}^{j/i} \end{array}$$
    (51) $$\begin{array}{cc} P_{k|k}^{j/i(P)} & =P_{k|k-1}^{j/i(P)} \end{array}$$

### 5.6. Event Triggering Condition

Two different event triggering algorithms will be considered in this proposed system. These two algorithms are paralleled together and make the final triggering decision. The sensor agent in Figure 3 makes a decision between using the communication resources based on these two algorithms to improve the accuracy of prediction or to save the communication resources, but will lose some part of this accuracy in terms of a degenerated prediction performance.

The communication cost is considered the design parameter $\delta_{k}^{i}$ in the optimization problem (28), and it could be defined based on the use of bandwidth or energy in the communication system. Based on [8], the design of $\left\{\gamma_{k}^{i}\right\}$ and $\left\{u_{k}^{i}\right\}$ can be separated from each other in the optimization problem (28). Therefore, the triggering decision can be derived as

(52) $$\min_{\left\{\gamma_{k}^{i}\right\}}E\left[\sum_{k=0}^{M-1}\left(\gamma_{k}^{i}\delta_{k}^{i}+\left(1-\gamma_{k}^{i}\right)\left(E_{1|k}^{i}+\sum_{j\in N_{N}\backslash \mathrm{not}\left[i\right]}E_{2|k}^{j/i}\right)\right)\right],$$

where we assume that $\delta_{k}^{i}$ is known for each agent. By solving the optimization problem (52), the event triggering law is obtained as follows:

(53) $$\;\mathrm{at}\;\mathrm{time}\;k:\;\gamma_{k}^{i}=1\Leftrightarrow E\left[E_{1|k}^{i}+\sum_{j\in N_{N}\backslash \mathrm{not}\left[i\right]}E_{2|k}^{j/i}\right]\geq \delta_{k}^{i}.$$

The expected values of each cost function are non-negative, so the trigger law can be rewritten as follows

(54) $$\;\mathrm{at}\;\mathrm{time}\;k:\;\gamma_{k}^{i}=1\Leftrightarrow \left\{\begin{array}{c} E\left[E_{1|k}^{i}|I_{k}^{i}\right]\geq \delta_{k}^{i}\\ \mathrm{or}\\ E\left[\sum_{j\in N_{N}\backslash \mathrm{not}\left[i\right]}E_{2|k}^{j}|I_{k}^{j/i}\right]\geq \delta_{k}^{i} \end{array}\right..$$

## 6. Illustrative Application and Simulation Platform

In this section, a platoon of vehicles will be described as an application that uses the proposed event triggering algorithm in a synchronization problem. Then, the models of the platoon network topology and longitudinal vehicle dynamics are represented. Furthermore, a simulation platform for simulating the platoon of vehicles in a wireless network is introduced.

### 6.1. Application

To illustrate the behavior of the PET algorithm, we consider a vehicle platoon control problem as a synchronization problem of a wireless networked dynamical system. In this configuration, the first vehicle is considered as a platoon leader, and a set of followers’ vehicles (two vehicles) interact through a communication network. Vehicle platooning is regarded as a multi-agent linear time-invariant system with dynamics interaction (1). They need to update the states of other agents in their control process through wireless communication. These vehicles are controlled so that the dynamics of the followers converge towards the dynamics of the leader.

### 6.2. Modeling of the Vehicle Platooning

For vehicle platooning applications, vehicle longitudinal dynamics can be represented by a second order linear time invariant system. The state of each vehicle $x_{i}^{T}\left(t\right)=\left[v_{i}\left(t\right),s_{i}\left(t\right)-s_{i-1}\left(t\right)\right],i=2,\ldots ,N_{N}$ except the leader, where *v* is speed and *s* is absolute position and *a* is acceleration, is considered as the control input.

**Figure 4:**
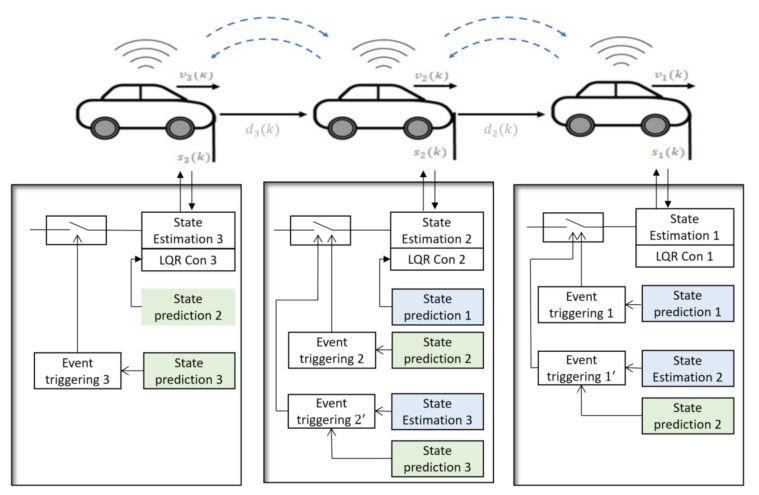
Schematic of vehicle platooning with the proposed event triggering algorithm.

The architecture of the vehicle platoon with the proposed event triggering algorithm is shown in Figure 4. Communication between the vehicles is required to control the distance between the vehicles. We assume that when an event is triggered in one vehicle, the data will be sent to the rear vehicle that interacts with it and the front vehicle that gets interaction from it. However, this information may not reach the recipient agents due to the possibility of the packet drop, so we use this proposed event triggering algorithm to improve the prediction of the information.

The longitudinal vehicle dynamics of the *i*th-follower in vehicle platooning, subjected to its interaction with the front vehicle can be obtained as follows:

(55) $$\left[\begin{array}{c} a_{i}\\ v_{i}-v_{i-1} \end{array}\right]=\left[\begin{array}{cc} 0 & 0\\ 1 & 0 \end{array}\right]\left[\begin{array}{c} v_{i}\\ s_{i}-s_{i-1} \end{array}\right]+\left[\begin{array}{c} 1\\ 0 \end{array}\right]a_{i}+\left[\begin{array}{cc} 0 & 0\\ -1 & 0 \end{array}\right]\left[\begin{array}{c} v_{i-1}\\ s_{i-1}-s_{i-2} \end{array}\right],$$

which is the continuous-time state space model, and can be converted into a discrete-time model by using the Euler method with the sampling time $T_{s}$:

(56) $$\begin{array}{cc} x_{k}^{i} & =A_{i}x_{k-1}^{i}+B_{i}u_{k-1}^{i}+w_{k}^{i}+N_{i-1}{\overset{ˇ}{x}}_{k-1}^{i-1}, \end{array}$$

(57) $$\begin{array}{cc} y_{k}^{i} & =C_{i}x_{k}^{i}+v_{k}^{i}, \end{array}$$

where the system matrices in this model are:

(58) $$A_{i}=\left[\begin{array}{cc} I & 0\\ T_{s} & I \end{array}\right],\;B_{i}=\left[\begin{array}{c} T_{s}\\ 0 \end{array}\right],\;N_{i-1}=\left[\begin{array}{cc} 0 & 0\\ -T_{s} & 0 \end{array}\right].$$

The model in (56) is an illustrative example of the general model (22), in which each vehicle only interacts with a vehicle in front. The first car is the leader in this configuration and does not have any interaction from the front.

### 6.3. Platoon Control Objectives

The platoon control objectives are to control the convergence of all followers’ dynamics with the leader dynamics and maintain the vehicles’ desired distance. We designed an LQR as a decentralized controller for each vehicle. The liner state-space model in this problem includes the vehicle speed and their relative distances $x_{k}^{i}{}^{T}=\left[v_{k}^{i},s_{k}^{i}-s_{k}^{i-1}\right],i=2,3$, and $v_{k}^{1}$ is the leader’s speed, therefore, if a constant spacing is considered as a desired space between vehicles and the leader speed is considered as a desired speed for the platoon $x_{ref,k}^{i}{}^{T}=\left[v_{ref,k}^{i},d_{ref,k}^{i}\right]$, then the LQR controller causes the states of each vehicle to follow the references.

### 6.4. Simulation Platform

The simulation application was developed in TrueTime, a Matlab/Simulink-based simulator for network and embedded control systems. This simulator can co-simulate controller task execution in real-time kernels, network transmission, and continuous plant dynamics. First, the dynamic process of each vehicle is modeled in Simulink, and then they make an internal communication between each other with the use of TrueTime’s kernel.

### 6.5. Cost Function about Resource Utilization

In order to analyze the network usage, a cost function $J_{net}$ is proposed as the resource utilization index. Let us define the number of events triggered based on the algorithm A between the vehicles as $NOET_{A}$, which will be compared with the total number of events that might be possible (TNEP) based on the sampling time in the communication network. In this way, the resource utilization (cost function $J_{net}$) can be expressed as

(59) $$J_{net}=\frac{NOET_{A}}{TNEP}\cdot 100\%.$$

This cost function will be used to evaluate the proposed event triggering algorithm in this simulation. The study will compare both approaches based on this cost function: PET $E_{1|k}^{i}+E_{2|k}^{i}$ versus general event triggering, only $E_{1|k}^{i}$.

## 7. Simulation Results and Discussion

The simulation of vehicle platooning through the WNCS was developed in the TrueTime simulator with three vehicles over the simulation horizon of 200 samples, and 100 simulation runs. All simulations were performed on a modified network protocol according to the simulation parameter for a wireless network provided in Table 2. A power control technique was used in TrueTime to adjust the transmission power on each vehicle by getting feedback from the receiver. Due to the mobility of vehicles in an urban area, there exists a range for path loss exponent in such environments, however, we considered a constant value in the upper range for this parameter. In addition, the bandwidth is limited to 80 kps, due to the feature of event triggering on resource utilization and the retry limit parameter is set to zero, which means that we simulated a wireless network protocol without sending and receiving acknowledgment, such as the UDP protocol. Furthermore, it is assumed that if the delay is more than the event sampling period, it is considered as a message drop and if it is less than the event sampling period, it can be ignored.

The proposed event triggering algorithm was simulated, and each vehicle decided to broadcast its states based on (54). The main goal of this part is to present the main benefit of the PET algorithm that can combat the packet drop effects, compared to the standard event triggering algorithm on a multi-agent system, without considering packet drop (see [14,15]).

For this simulation, the desired inter-vehicle spacing was set to $d_{ref,k}=10$ m. The initial states for each vehicle were chosen randomly at time t=0, and the platoon drives for 20 s. The first vehicle starts to move as the leader to reach a constant speed of $40\;\mathrm{km}\;h^{-1}$. The external disturbance is considered an additional load on the leader, which slows down its speed, starting at t=8, and the leader’s controller tries to compensate for this disturbance. However, it influences the followers’ dynamics.

**Figure 5:**
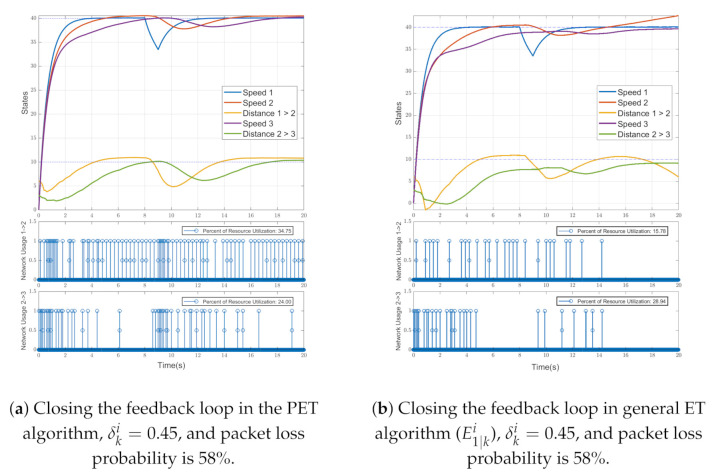
The graphs show, from top to bottom, the states of vehicles in platoon (speeds ($\mathrm{km}\;h^{-1}$), relative distance (m)), the network usage between vehicles 1→2, and 2→3. The value of the resource utilization is mentioned on the graphs as previously defined.

For both event triggering algorithms, a 58% packet drop is considered. The highest probability value for the packet drop that the PET algorithm could converge this multi-agent system is 65%. For the values above this percentage, even the PET algorithm cannot converge the system. We first considered the PET results by using the event triggering conditions (54) shown in Figure 5a with a constant $\delta_{k}^{i}=0.45$. This consists of the state estimation of all vehicles in the platoon at the top subplot. At the middle and bottom, subplots are binary variables that show the network usage between vehicles 1→2 and 2→3, respectively. Moreover, 0 means that the network is not being used, and 1 means that the network is being used. Figure 5a shows that even with 58% packet drops, the proposed event triggering algorithm can converge the followers to the leader motion and maintain the desired inter-vehicular distance.

Figure 5b shows the results obtained by the general event triggering algorithm. As shown in the top subplot, the collision occurred in the early moments of the simulation between the vehicles, and the general event triggering algorithm ($E_{1|k}^{i}$) could not converge the followers’ dynamics to the leader. In this algorithm, the cost function of using resource utilization is decreased compared to the PET algorithm, but the system is not stable. As a result, it could not converge to the platoon’s dynamics. Therefore, the proposed event triggering algorithm can converge the platoon of vehicles even in a harsh industrial environment with a 58% probability of packet drops by an increasing network resource utilization, compared to the general ET that can be seen in Table 3. In contrast, the general ET cannot converge the system anyway. In PET, the middle and bottom subplots show the increase in network usage compared to the general ET algorithm, but this is the cost paid to maintain system convergence.

**Figure 6:**
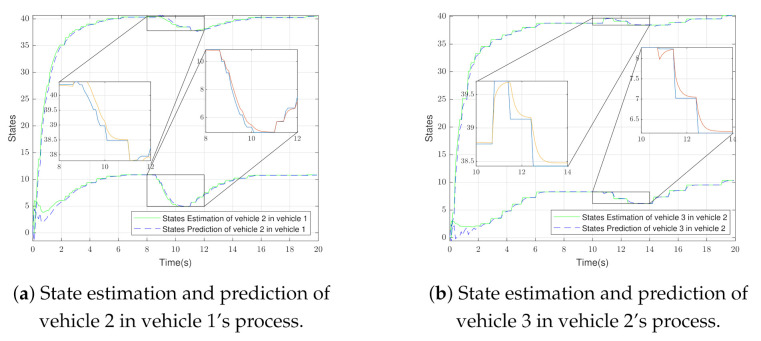
Distributed state estimation and prediction in PET.

The state estimation and prediction of the second vehicle in the process of the first vehicle and the state estimation and prediction of the third vehicle in the process of the second vehicle are shown in Figure 6a,b, respectively. As mentioned in Figure 4, to implement the PET algorithm, the state estimation and prediction of the second vehicle are required in the first vehicle’s control process, in addition to the state estimation and prediction of the third vehicle are required in the second vehicle’s control process. When a packet drop has happened in the state broadcasting of the first vehicle, the discrepancy between the state estimation and prediction of the second vehicle, which interacts with the first vehicle, increases and causes a new event triggered. This process was done in the control routine of the first vehicle.

**Figure 7:**
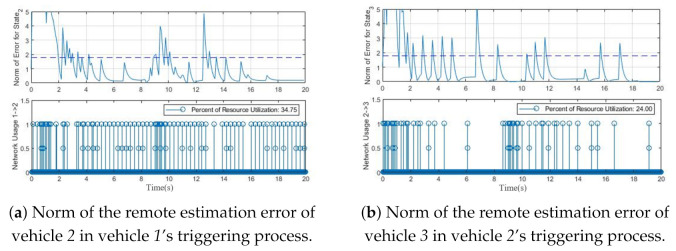
Remote estimation error in PET.

Finally, Figure 7a,b depict the norm of error between the state estimation and prediction of the second and third vehicle, respectively. As expected, when the remote estimation error $e_{k}^{i}=∥{\hat{x}}_{k}^{j}-{\overset{ˇ}{x}}_{k}^{j}∥$ passes the threshold, a new event will be generated. The density of events will be triggered when the remote estimation error exceeds the threshold, which is obviously visible in these figures. Hence, the proposed PET algorithm can be used in the multi-agent triggering system with a high probability of packet drop and guarantees the dynamics convergence of all agents.

## 8. Conclusions

This paper studied the distributed feedback control in a multi-agent system over a WNCS framework. DEBSE was used to design the parallel event triggering algorithm in WNCS to face high packet drop probability conditions such as those of an industrial environment and maintain control performance on consensus problems in the multi-agent system at the desired level. Integrating ET algorithm in WNCS enables a significant reduction in network resource usage. The proposed PET algorithm slightly increases the utilization of network resources compared to ET, but maintains satisfactory control performance in multi-agent consensus problems under a harsh packet drop conditions.

## Figures and Tables

**Table 1:** Summary of main variables and notations used in this paper.

Symbol	Definitions
$A_{i},B_{i},N_{i},C_{i},Q_{i},R_{i}$	Dynamic system parameters for agent *i*
$N_{N}$	Set of all positive integers {1,2,…,N}
$U_{k}^{i}$	Set of all inputs on agent *i* until time *k*
$Y_{k}^{i}$	Set of all measurements on agent *i* until time *k*
$F_{i}$	Optimal feedback gain corresponding to agent *i*
$x_{k}^{i}$	State of agent *i*
${\hat{x}}_{k}^{i}$	Local state estimate of agent *i*
${\overset{ˇ}{x}}_{k}^{i}$	Remote state estimate (predict) of agent *i*
${\hat{x}}_{k}^{j/i}$	Remote state estimate of agent *j* in agent *i*
${\overset{ˇ}{x}}_{k}^{j/i}$	Remote state predict of agent *j* in agent *i*
$\gamma_{k}^{i}\in \left\{0,1\right\}$	Communication decision variable for agent *i*
$\Gamma_{k}^{i}$	Set of communication decision $\Gamma_{k}^{i}=\left\{\gamma_{1}^{i},\ldots ,\gamma_{k}^{i}\right\}$
$I_{k}^{i}$	The information set available to agent *i* at time *k*
$\nu_{k}^{j/i}$	Packet dropping random variable, from agent *j* to *i*
$\lambda_{j/i}$	Probability of successfully receiving the packet, from agent *j* to *i*
$I_{k}^{j/i}$	The information set available to agent *i*, from agent *j*
$\Xi_{k}^{i}$	All information gathered from sensors on agent *i*
$P^{\left(E\right)}$	Predicted or updated estimate covariance
$P^{\left(P\right)}$	Predicted or updated predict covariance
$E\left[X_{1}|X_{2}\right]$	Expected value of $X_{1}$ cond. on $X_{2}$

**Table 2:** Simulation parameters for wireless network.

Parameters	Values
Network type	802.11b (WLAN)
Data rate	80 kps
Minimum frame size	272 bits
Maximum transmit power	20 dbm
Receiver signal threshold	−48 dbm
Path loss exponent	3.5
Retry limit	0

**Table 3:** $J_{net}$ index for the simulation.

Index	Vehicle 1→2	Vehicle 2→3
PET	34.75%	24%
ET	28.94%	15.78%

## Data Availability

The data generated in the simulation part are available on request from the corresponding authors.

## Abbreviations

The following abbreviations are used in this manuscript:

WNCSs	Wireless Networked Control Systems
ETC	Event-Triggered Control
TTC	Time-Triggered Control
KF	Kalman Filter
DEBSE	Distributed Event-Based State Estimation
PET	Parallel Event Triggering
NOET	Number of Events Triggered
TNEP	Total Number of Events that Might Be Possible
